# Acute exacerbations of chronic obstructive pulmonary disease: in search of diagnostic biomarkers and treatable traits

**DOI:** 10.1136/thoraxjnl-2019-214484

**Published:** 2020-03-26

**Authors:** Alexander G Mathioudakis, Wim Janssens, Pradeesh Sivapalan, Aran Singanayagam, Mark T Dransfield, Jens-Ulrik Stæhr Jensen, Jørgen Vestbo

**Affiliations:** 1 Division of Infection, Immunity and Respiratory Medicine, University of Manchester, Manchester, UK; 2 North West Lung Centre, University Hospital of South Manchester NHS Foundation Trust, Manchester, UK; 3 Respiratory Division, Department of Clinical and Experimental Medicine, University Hospital Leuven & KU Leuven, Leuven, Belgium; 4 Section of Respiratory Medicine, Department of Medicine, Herlev and Gentofte Hospital, University of Copenhagen, Hellerup, Denmark; 5 National Heart and Lung Institute, Imperial College London, London, UK; 6 Division of Pulmonary, Allergy and Critical Care Medicine, University of Alabama School of Medicine, Birmingham, Alabama, USA; 7 Department of Clinical Medicine, University of Copenhagen Faculty of Health and Medical Sciences, Copenhagen, Denmark; 8 PERSIMUNE&CHIP: Department of Infectious Diseases, Rigshospitalet, Copenhagen, Denmark

**Keywords:** COPD Exacerbations

## Abstract

Acute exacerbations of chronic obstructive pulmonary disease (COPD) are associated with a significant mortality, health and economic burden. Their diagnosis, assessment and management remain suboptimal and unchanged for decades. Recent clinical and translational studies revealed that the significant heterogeneity in mechanisms and outcomes of exacerbations could be resolved by grouping them etiologically. This is anticipated to lead to a better understanding of the biological processes that underlie each type of exacerbation and to allow the introduction of precision medicine interventions that could improve outcomes. This review summarises novel data on the diagnosis, phenotyping, targeted treatment and prevention of COPD exacerbations.

## Introduction

Acute exacerbations, punctuating the natural history of chronic obstructive pulmonary disease (COPD), are associated with significant mortality as well as health and socioeconomic burden.^1–3^ They are the main drivers of the poor outcomes of COPD, which is consequently ranked as a leading cause of death and disability globally.^1–3^ It is estimated that every year 22%–40% of all patients with COPD experience at least one moderate or severe exacerbation, while 9%–16% experience more than one.^4 5^ As a result, exacerbations are responsible for one in eight emergency hospital admissions in the UK; an enormous number, considering that the 3-month mortality rate of a hospitalised exacerbation exceeds 15%.^1 2 6^ Patients experiencing frequent exacerbations have worse quality of life,^7^ accelerated lung function decline^8^ and are at increased risk of future exacerbations, myocardial infarctions, cerebrovascular events and mortality.^9–12^

With a definition, diagnostic criteria and treatment strategies that remain insufficient and unchanged for decades,^1–3^ COPD exacerbations represent a major, unaddressed global health need. More specifically, their definition and diagnostic criteria are solely based on clinical characteristics, which lack specificity. The same symptoms are present in stable COPD or they can result from other respiratory or cardiac diseases.^13 14^ Therapeutically, exacerbations are approached as a single entity and treated uniformly with the administration of bronchodilators, systemic corticosteroids and commonly antibiotics.^2 3^ In reality, they are heterogeneous, characterised by diverging underlying mechanisms, outcomes and treatment needs. More specifically, only about 30%–50% of all exacerbations are associated with enhanced airway eosinophilic inflammation and appear to respond to systemic corticosteroids, whereas around 50% are triggered by bacterial infections and may respond to antibiotics.^15–17^ Finally, around 30% that are triggered by viruses^13 14^ are currently not treated etiologically, despite the availability of potentially beneficial treatments. Importantly, the heterogeneity of COPD extends beyond exacerbations. Therefore, subgroups of patients may be predisposed to specific subtypes of exacerbations, suggesting a potential role of precision medicine in exacerbations prevention.

This review summarises novel data on the diagnosis, phenotyping, targeted treatment and prevention of COPD exacerbations. A detailed description of the epidemiology, mechanisms, impact or outcomes of pneumonia, in patients with COPD, or of disease entities that could mimic COPD exacerbations was considered beyond the scope of this review.

## Current definitions and diagnostic criteria

The development of an accurate definition and diagnostic criteria is impeded by the complexity and heterogeneity that characterises COPD exacerbations and our limited insight into their underlying mechanisms. As a result, all available definitions are solely based on clinical symptoms and have important limitations. An exacerbation is commonly described as an acute deterioration of the respiratory symptoms of patients with underlying COPD that results in additional therapy (table 1). These definitions have poor specificity, as numerous other respiratory or non-respiratory diseases may have a similar presentation.^18^ Their sensitivity is also poor, as patients who experience increased symptoms often do not seek medical advice and do not receive additional treatments.^7 19^ Most importantly, these definitions rely on patients’ and clinicians’ perceptions of the symptoms for diagnosis and severity assessment, without providing guidance for a standardised approach.

**Table 1:** Frequently used definitions and diagnostic criteria for COPD exacerbations

GOLD definition	‘Acute exacerbations are episodes of acute worsening of the respiratory symptoms of patients with COPD, that result in additional therapy^1^
Consensus conference 2002 (Rodriguez-Roisin) definition	‘In patients with underlying COPD, exacerbation is an acute sustained symptoms worsening from the stable state that is beyond normal day-to-day variation and necessitates a change in regular medications^13^
COPD-X (Australian and New Zealand Guidelines)	‘A COPD exacerbation is characterized by a change in the patient’s baseline dyspnea, cough, and/or sputum that is beyond normal day-to-day variations, is acute in onset, and may warrant a change in regular medication or hospital admission^20^
NICE (UK Guidelines)	‘An exacerbation is a sustained worsening of the patient’s symptoms from their usual stable state which is beyond day-to-day variations and is acute in onset. Commonly reported symptoms are worsening breathlessness, cough, increased sputum production and change in sputum colour. The change in these symptoms often necessitates a change in medication^2^
Anthonisen criteria	‘In patients with underlying COPD, an exacerbation is an acute, sustained deterioration of at least two of the following symptoms: Increased sputum volume; increased sputum purulence; breathlessness’^23^
Modified Anthonisen criteria	‘In patients with underlying COPD, an exacerbation is an acute sustained deterioration of at least two of the following major symptoms or at least one major and one minor symptom. Major symptoms: Increased sputum volume; increased sputum purulence; breathlessness. Minor symptoms: cough; wheeze; nasal discharge; sore throat; pyrexia’^24^
EXACT criteria	‘The EXACT patient-reported diary is a patient-reported outcome measure developed to identify COPD exacerbations, by quantifying daily the intensity of the following symptoms: congestion, cough, sputum production, sputum thickness, chest discomfort, chest tightness, and breathlessness. An exacerbation is defined by an increase of at least 12 points for two consecutive days or an increase of at least 9 points for at least three consecutive days’^25^

COPD, chronic obstructive pulmonary disease; EXACT, The Exacerbations of Chronic Pulmonary Disease Tool; GOLD, Global Initiative for Chronic Obstructive Lung Disease; NICE, National Institute for Health and Care Excellence; NICE, The National Institute for Health and Care Excellence.

The Australian and New Zealand (COPD-X) and UK (National Institute for Health and Care Excellence) guidelines also define exacerbations on the basis of symptoms, but administration of additional treatments is not a prerequisite.^2 20^ This approach may be more sensitive, but at the expense of an even lower specificity.

Clinical research studies often use more strict diagnostic criteria, aiming to select a more homogeneous group of events.^21 22^ In the 1980s, Anthonisen *et al* described exacerbations as acute, sustained deterioration of at least two symptoms of sputum volume, sputum purulence and breathlessness.^23^ These criteria were considered less sensitive to non-infective events and for this reason, a modified version was proposed, describing exacerbations as an acute deterioration of at least one of the aforementioned major criteria and either a second major or one minor criterion(cough, wheeze, nasal discharge, sore throat or fever).^24^ Daily monitoring of symptom intensity compared with baseline has also been suggested as a more accurate method to capture all exacerbations. Examples include the Exacerbations of Chronic Pulmonary Disease Tool (EXACT) questionnaire or the London COPD diary cards.^7 25^ While daily monitoring is currently used mainly in clinical research, the development of effective mobile applications could facilitate their introduction to clinical care.^26^

The characteristics and outcomes of the episodes that are captured when using each of these definitions or diagnostic criteria for exacerbations are very different. The modified Anthonisen criteria used in the London COPD cohort study led to the identification of twice as many exacerbations compared with those leading to healthcare utilisation by patients.^27^ Unfortunately, it is still unclear which of these episodes are associated with increased risk of death, disability, poor quality of life, disease progression or cardiovascular events, and require more aggressive management. Thus, there is an urgent need for accurate diagnostic and prognostic biomarkers to complement clinical characteristics.

## Diagnostic and prognostic biomarkers

The National Institutes of Health (NIH) working group defined a biomarker or biological marker as ‘a characteristic that is objectively measured and evaluated as an indicator of normal biological processes, pathogenic processes or pharmacologic responses to a therapeutic intervention’.^28^ In the context of COPD exacerbations, the role of biomarkers encompasses different areas. They can be applied as a diagnostic tool for early detection of events, as staging tools to classify disease severity and/or identify important subgroups, as prognostic tools to predict clinically important outcomes and, probably most importantly as therapeutic tools to identify treatment indications and monitor response. Ideally, a biomarker for COPD exacerbations is mechanistically linked to the acute burst of airway inflammation which it detects with both high negative and high positive predictive value. It should be amendable to existing therapeutic interventions (treatable trait)^29^ and serve as a surrogate endpoint for prognostic purposes. Moreover, for broad clinical implementation, a biomarker should be obtained non-invasively, highly reproducible and preferentially available at low cost. As cardiac troponin T covers most of these demands in the context of acute myocardial infarction, for many years the challenge within COPD exacerbations has been in searching for an equally well-performing biomarker. However, it seems generally accepted that due to the heterogeneity of exacerbations, such a ‘global’ marker most likely does not exist.^30 31^

From the Evaluation of COPD Logitudinally to Identify Predictice Surrogate End-points (ECLIPSE) study, following 2138 patients with COPD for 3 years, it was demonstrated that number of previous exacerbations, history of heartburn or reflux, forced expiratory volume in 1 s (FEV_1_) and quality of life were the most important determinants for future exacerbation risk prediction.^9^ Importantly, several blood biomarkers, such as C-reactive protein (CRP), fibrinogen, surfactant, cytokines, white blood cell differentiation, did not significantly improve risk prediction when history of exacerbations were adjusted for. A more recent extensive analysis on 119 blood biomarkers from the COPD Genetic Epidemiology study (COPDGene) and the Subpopulations and Intermediate Outcome Measures in COPD study (SPIROMICS) cohort confirmed that blood markers added little to the predictive value of clinical covariates for exacerbations.^32^ An accompanying editorial even stated that the search for a single blood biomarker of exacerbation frequency had come to an end.^33^ It is noteworthy that the majority of participants in these studies came from secondary respiratory centres. In 6574 subjects with COPD identified in general population studies in Copenhagen, simultaneous elevated levels of CRP, fibrinogen and total white blood cell count associated with higher risk for exacerbations, particularly in the clinical subgroup of patients at risk because of exacerbation history or poor FEV_1_.^34^ Moreover, an accurate imaging biomarker was identified in an analysis from the COPDGene and ECLIPSE studies. A ratio of the diameter of the pulmonary artery to that of the aorta exceeding one and suggesting pulmonary hypertension was independently associated with a threefold increase in the risk of exacerbations.^35^ Physiological markers, such as increased respiratory impedance, were also predictive of future exacerbations.^36^

When focussing on the role of biomarkers to detect an ongoing acute exacerbation, Noell *et al* performed a multilevel network analysis including blood and sputum biomarkers on top of a detailed clinical characterisation.^37^ Unsurprisingly, they found that a combination of elevated CRP, neutrophils and increased levels of breathlessness was the best panel to discriminate between acute exacerbation and stable state. More importantly, another group evaluating the association of exacerbations with lung tissue injury, demonstrated altered circulatory levels of several respiratory extracellular matrix turnover biomarkers with diagnostic potential, in two independent patient cohorts.^38 39^ These biomarkers, being mechanistically linked to lung tissue injury, may be able to identify those events that are associated with future risk of poor outcomes, although this will need to be further evaluated. Such patterns of biomarkers of lung tissue injury have the potential to revolutionise the management of exacerbations the same way that troponin revolutionised the management of acute cardiac chest pain by differentiating myocardial infarctions from simple episodes of angina.^40^ Non-circulatory biomarkers have also been evaluated. Interestingly, exploratory studies identified differences in the exhaled breath profiles of patients with COPD during stable disease state versus exacerbations, suggesting a potential diagnostic role.^41 42^Many individual clinical features and scorings based on multiple, independent and relevant clinical characteristics have been validated to predict outcomes of the acute event. Dependent on the outcome (within hospital mortality, 90-day post-onset mortality, readmission or time to the subsequent event) different clinical features will dominate in the prediction tool. Unfortunately, the European COPD audit comprising clinical data of more than 13 000 hospital admissions for exacerbations was not able to evaluate the added value of blood or sputum biomarkers on prognostication.^43^ Nevertheless, some single-centre studies highlight the important prognostic value of eosinopenia, acidaemia^44^ and the cardiac blood markers troponin T and N-terminal(NT)-pro-Brain Natriuretic Peptide (NT-proBNP) when corrected for clinical covariates.^45 46^ Several circulatory biomarkers of extracellular matrix turnover were found more deranged in severe, compared with moderate exacerbations, suggesting a potential prognostic value.^39^ Neural respiratory drive, a physiological marker, also appears predictive of treatment response and the long-term outcomes of exacerbations.^47 48^ For a more comprehensive insight on the prognostication role of blood or sputum markers measured at the onset of exacerbations, discharge or end of medical intervention, larger multicentre studies are needed.

**Figure 1:**
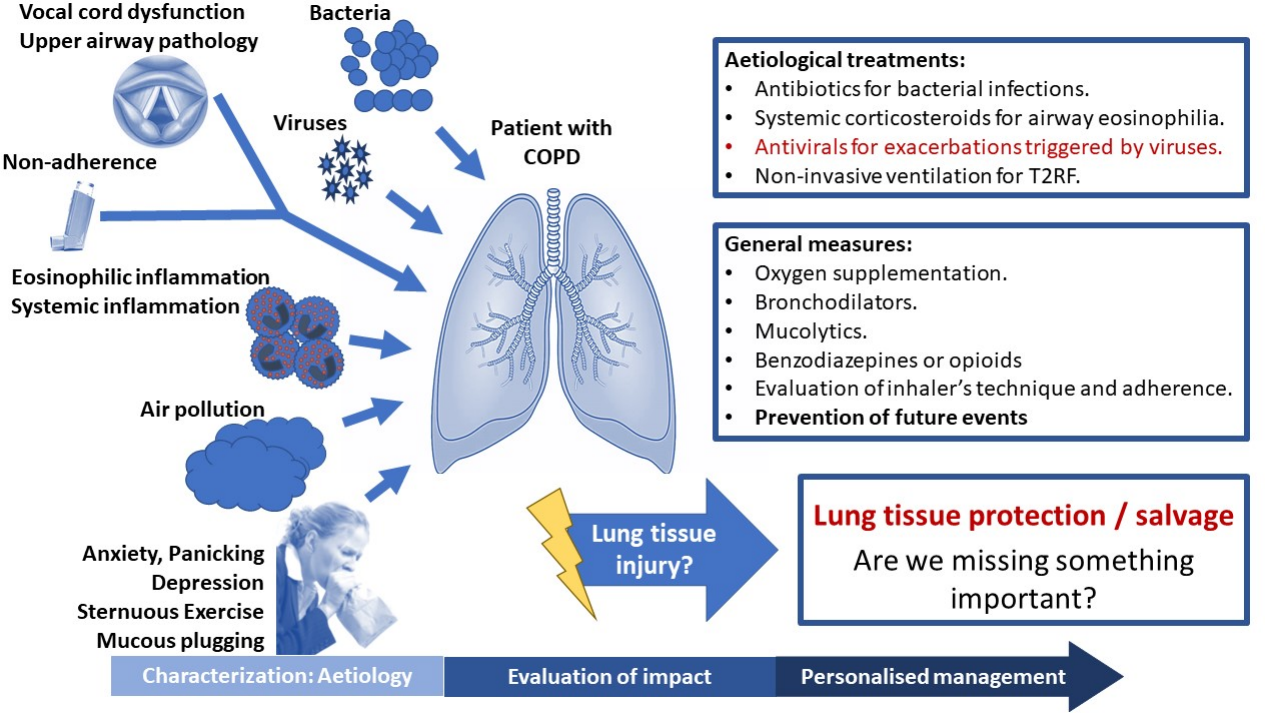
Characterisation and aetiological treatment of acute exacerbations of chronic obstructive pulmonary disease.

A possible way of facilitating biomarker discovery in COPD exacerbations could be to better identify and characterise the subtypes of exacerbations. Classification of exacerbations by their causative agent, such as bacterial infections, viral infections or enhanced airway inflammation, appears to be the most promising (figure 1).^15 17^ With an unbiased cluster analysis based on blood and sputum biomarkers, Bafadhel and colleagues demonstrated exacerbations of the same aetiology (bacteria, viruses, enhanced eosinophilic inflammation or pauci-inflammatory) exert similar inflammatory patterns. In line with these studies, several groups explore the mechanisms and potential biomarkers of each of these types of exacerbations. Exacerbations triggered by bacteria, viruses or enhanced eosinophilic inflammation are discussed below.

## Bacteria and COPD exacerbations

Bacteria are frequently identified in sputum culture in over 50% of exacerbations, especially during winter months, but high detection rates (>25%) also occur during stable disease.^15 17 49–53^ Therefore, the identification of bacteria during an exacerbation cannot establish bacterial aetiology. In the absence of experimental bacterial challenge studies in COPD, it remains unconfirmed whether bacteria can directly trigger exacerbations. The various mechanisms regarding how bacteria might trigger exacerbations are discussed below.

Studies have examined whether an increase in lung bacterial loads could induce exacerbation. Several large observational studies have reported increased bacterial loads at exacerbation compared with stable state; Rossell *et al* confirmed these findings using protected brush speciments.^52^ Conversely, Sethi *et al* showed no difference in sputum concentrations of potentially pathogenic bacteria *Haemophilus influenzae* and *Moraxella catarrhalis* from stability to exacerbation and also showed inverse relationships between concentrations of *Streptococcus pneumoniae* and *Haemophilus parainfluenzae* with exacerbation occurrence.^54^ Thus, evidence to support outgrowth of specific pathogens triggering exacerbation remains limited.

An alternative concept is that acquisition of a new strain of bacterium could be a potential mechanism of COPD exacerbation. Sethi *et al* used molecular typing to show that detection of a new, previously unencountered, bacterial strain was associated with a significantly increased risk of exacerbation (relative risk 2.15; 1.83–2.53; p<0.001).^55^ Specifically, acquisition of new strains of *H. influenzae*, *M. catarrhalis* and *S. pneumoniae* was all shown to be significantly associated with increased risk of exacerbation.^55^ Importantly, similar associations were not observed when considering cultured bacteria without strain information in this study. In a follow-up study from this group, clinical and inflammatory responses in patients with new bacterial strain acquisition were evaluated.^56^ New strain exacerbations were characterised by greater symptom scores, had a greater increase in airway inflammation including augmentation of the pro-inflammatory cytokine tumour necrosis factor-α and neutrophil elastase when compared with pathogen-negative exacerbations. These data were supported in a separate study by Chin *et al*, where strains of *H. influenzae* isolated during exacerbations induced greater inflammatory responses in human airway epithelial cell cultures and mouse infection models than strains isolated during clinical stability.^57^

Lung microbiota alterations have also been incriminated as potential causes of exacerbations. Modern molecular sequencing techniques have revealed that the healthy respiratory tract is colonised by microbiota consisting of complex bacterial communities.^58^ The microbiota in COPD is broadly characterised by an outgrowth of the Proteobacteria phylum and an increase in proportion of *Streptococci* and *Staphylococci* within the Firmicutes phylum^58–60^ Our knowledge of the dynamic shifts that occur within the lung microbiota during exacerbations is limited but recent studies have shed some light on this topic.^61–64^ Mayhew *et al* showed in the Acute Exacerbations and Respiratory Infections in COPD (AERIS) study that expansion of Proteobacteria (*Moraxella*) within the microbiota are observed at exacerbation.^63^ Wang *et al* reported data from the COPDMAP study showing that microbial dysbiosis was present in 41% of exacerbations and was associated with increased acute FEV_1_ decline and increased COPD Assessment Test (CAT) scores.^62^ Further studies are needed to understand if and how shifts in the microbiota may lead to exacerbation and to understand the immune mechanisms responsible for these changes.

An alternative mechanism through which bacteria could trigger COPD exacerbation is following an initial virus infection. Studies of naturally occurring and experimentally induced exacerbations have demonstrated the occurrence of secondary bacterial infections following an initial rhinovirus infection^64–66^ Neutrophil elastase-mediated cleavage and reduction of the antimicrobial peptide Secretory Leukocyte Protease Inhibitor (SLPI) is believed to be important mechanistically^65^ and may be worsened by chronic use of inhaled corticosteroids (ICSs).^67^

Despite the fact that the role of bacterial infection as a trigger for exacerbations of COPD is unconfirmed, antibiotic use remains widespread:>80% in secondary care and around 50% in primary care.^68 69^ The Global Initiative for Chronic Obstructive Lung Disease (GOLD) documents state that antibiotic use for COPD exacerbations is category B evidence (few randomised studies exist, small in size and heterogeneous populations).^1^ In particular, there are very few placebo-controlled trials. A major limitation with current approaches to exacerbation management is the lack of a reliable rapid biomarker of bacterial infection to facilitate more targeted antibiotic prescribing. Older methods such as the Anthonisen criteria (symptom complex to identify patients with greater likelihood of bacterial infection)^23^ are likely to be insensitive. Newer biomarkers are currently being clinically validated. The use of CRP to guide antibiotics administration has been evaluated in two recent randomised controlled trials (RCTs). The C-Reactive Protein Testing to Guide Antibiotic Prescribing for COPD Exacerbations (PACE) study involved 653 patients with moderate exacerbations,^70^ while the CRP-guided antibiotic treatment in acute exacerbations of COPD in hospital admissions (CATCH) trial randomised 101 patients with severe exacerbations.^71^ In both trials, use of CRP led to a modest decrease in antibiotics use (20.4% and 15.5% absolute decrease), without any adverse impact on the clinical outcomes. Procalcitonin-guided antibiotic administration has been evaluated in several RCTs. A recent meta-analysis including data from eight RCTs and 1062 patients suggested procalcitonin can decrease the proportion of patients with severe COPD exacerbations receiving antibiotics by 45% (absolute decrease of 28%), without adversely affecting clinical outcomes.^16^ It concluded that larger RCTs are needed to confirm these findings.^16^ Another RCT tested the hypothesis that knowledge of respiratory viruses screening findings could help clinicians reduce antibiotic administration.^72^ Not unexpectedly, this RCT did not show any evidence of reduction in antibiotics use. This biomarker is neither sensitive, since antibiotics are required in cases where bacteria and viruses coexist, nor specific, since exacerbations triggered by eosinophilic inflammation may also test negative for viruses.

## Respiratory viruses and COPD exacerbations

Respiratory viruses are identified in 30%–50% of all COPD exacerbations.^15 17 66 73^ However, they are also identified in >10% of patients during stable disease state, at any given time.^66 74 75^ Most frequently detected viruses in COPD include rhinovirus, influenza and respiratory syncytial virus (RSV).^15 17 74 75^ In contrast to the uncertainty on how bacteria trigger exacerbations, experimental viral challenge studies have confirmed a direct causal link between viral infections and COPD exacerbations.^76 77^

Limited information is available about the impact of the presence of respiratory viruses on the outcomes of COPD. Presence of viruses is associated with worse clinical outcomes both in stable COPD and in acute exacerbations.^74 75^ In the East London COPD cohort, the presence of any virus during stable disease state was associated with 60% more frequent exacerbations, while their presence during exacerbations was associated with delayed recovery and a higher symptoms burden.^75^ However, another retrospective cohort analysis found a lower mortality rate in exacerbations associated with respiratory viruses compared with those testing positive for bacteria either based on culture or PCR of sputum, endotracheal aspirates or bronchoalveolar lavage.^78^ Different respiratory viruses may have different effects on the immune responses and thus distinct clinical outcomes, but most studies evaluate the presence of all viruses and data are scarce on the differential clinical impact of specific virus types. Occasionally, both bacteria and viruses are identified in exacerbations and their coexistence is associated with a longer length of hospital stay and a higher symptom burden.^79^

As COPD exacerbations triggered by viruses are characterised by more prolonged and burdensome symptoms, antiviral vaccines and treatments may be beneficial. Several RCTs and large real-life studies have demonstrated that influenza vaccination is highly effective in reducing the frequency of COPD exacerbations.^80 81^ Neuraminidase inhibitors are of proven benefit for the treatment of influenza; however, in clinical practice, influenza is underdiagnosed in exacerbations.^82^ In contrast to healthy adults, use of commercially available antivirals for exacerbations triggered by other viruses (such as RSV)^83^ might be beneficial, since viral infections are associated with a higher burden in patients with COPD. However, their use in RCTs or clinical practice is impeded by the absence of accurate diagnostic biomarkers for exacerbations triggered by viruses.

While modern molecular techniques allow rapid identification of the presence of respiratory viruses possible, this cannot establish viral aetiology of an exacerbation, since respiratory viruses are often present in stable COPD. An accurate biomarker that can accurately confirm viral aetiology of exacerbations is still lacking. Symptoms of upper respiratory tract infection or common cold may concur or precede exacerbations associated with viruses; however, diagnostic accuracy of this approach is limited.^66 73^ Serum IP-10 is raised in rhinovirus-positive exacerbations and strongly correlates with viral load.^84^ Viral load also correlates with the severity of upper respiratory tract symptoms.^84^ This is consistent with the natural history of acute respiratory viral infections in healthy subjects where initial replication in the respiratory tract leads to high viral load that peaks early in the course of disease, and may be followed by a prolonged period of viral shedding and lower viral loads.^85^ A similar pattern was also observed in experimental viral challenge studies in patients with COPD.^76 77^ Therefore, a surrogate marker of viral load could be useful diagnostically.

## Exacerbations characterised by enhanced eosinophilic inflammation in the airways

Airway inflammation in COPD includes the presence of various inflammatory cells such as neutrophils, CD8 +T lymphocytes, mast cells, eosinophils and macrophages.^86^ Until recently, COPD was considered a primarily neutrophil-mediated inflammatory disease. However, 20%–40% of patients with COPD have been found to have eosinophilic airway inflammation, both during stable disease state and exacerbations, even after excluding subjects with coexisting asthma.^15 87 88^ Interestingly, these patients exhibit better response to corticosteroid therapy in both stable COPD and during exacerbations.^89–91^ Blood eosinophil count correlates reasonably well with eosinophil levels in sputum and airways and could be used as a surrogate measure of airway eosinophilia in COPD.^88^

In stable COPD, a higher blood eosinophil count is associated with an increased risk of future exacerbations,^92 93^ especially eosinophilic exacerbations.^94^ It is also predictive of treatment response (exacerbation reduction) to ICSs.^87 95^

COPD exacerbations characterised by enhanced airway eosinophilic inflammation are generally milder, as they are associated with lower mortality^44^ and shorter length of hospital stay.^96^ Bacterial infections are rarely present, while the presence of viral infection in exacerbations characterised by airway eosinophilia remains controversial.^15 92 97^ Higher blood eosinophil count during exacerbations is predictive of clinical response to oral corticosteroids.^90 91^ An RCT evaluating 166 mostly moderate severity exacerbations demonstrated the safety of withholding systemic corticosteroids in those characterised by a blood eosinophil count of less than 2% of total white cell count at baseline.^90^ While studies evaluating stable COPD suggested a degree of variability in blood eosinophils over time,^98^ this did not appear to limit the results of this trial. However, use of additional eosinophil measurements to guide the administration of systemic corticosteroids may be preferable, especially for severe exacerbations. Indeed, the Corticosteroid Reduction in COPD (CORTICO-COP) trial demonstrated non-inferiority of a treatment protocol where daily eosinophil measurements were used to guide daily administration of systemic steroids for severe COPD exacerbations, compared with standard care.^91 99^ Based on this treatment protocol, which reduced cumulative prednisolone dose by 60%, corticosteroids were administered for a maximum of up to 5 days—on days when the blood eosinophil count was at least 0.3×10^9^ cells/L. In line with these RCTs, a post-hoc analysis of three clinical trials, using blood eosinophils to direct oral corticosteroid therapy for the treatment of COPD exacerbations, found increased treatment failure rates in patients with a blood eosinophil count ≥2% who did not receive prednisolone compared with patients who did (66% vs 11%).^100^ On the contrary, increased harm was observed in patients with blood eosinophil count of <2%, when treated with prednisolone. There remains an ongoing debate as to the appropriate eosinophil count threshold for risk stratification. This has been investigated in more depth in studies evaluating treatment of stable COPD with ICS. Several studies have reported a 2% eosinophil threshold, while others have focused on the absolute eosinophil counts. Based on data from the COPDGene and ECLIPSE studies, a consistent linear relationship between eosinophil counts and exacerbation risk was seen. In the same study, multivariable logistic regression models using different eosinophil count cut-offs found that a threshold of 0.3×10^9^ cells/L was associated with the greatest sensitivity (72.6 %) and specificity (66.0%) for identifying a risk of self-reported exacerbation of at least one event.^101^ The Copenhagen Lung Study showed an increase in exacerbations when blood eosinophilia count was above 0.34×10^9^ cells/l^94^. Overall, blood eosinophils represent a continuous biomarker and a threshold with perfect performance characteristics is very unlikely to be found.

Better characterisation of the eosinophilic COPD phenotype may allow the introduction of more targeted treatment strategies. This could limit the use of corticosteroids in a patient group with a low eosinophil count, an already vulnerable patient group.

## Precision medicine in the prevention of exacerbations

Prevention of exacerbations is a crucial therapeutic aim in stable COPD. Most available treatments address this to some extent, as demonstrated by RCTs of unselected patients with COPD and frequent exacerbations (summarised in the ERS/ATS guideline on the prevention of COPD exacerbations^102^ and the GOLD document.^1^

More personalised preventive approaches have not been tested yet. However, there is emerging evidence suggesting a potential role for such interventions. Characteristically, a recent report from the AERIS longitudinal cohort suggested that patients tend to experience either recurrent bacterial or recurrent eosinophilic exacerbations.^63^ This group of patients might benefit from different preventive strategies. Consistently, in the Study to understand mortality and morbidity in COPD (SUMMIT) trial, the addition of an ICS (fluticasone furoate) led to a reduction in the frequency of exacerbations that were treated with systemic corticosteroids alone or with both antibiotics and systemic corticosteroids, with treatment being decided by the responsible clinician.^103^ On the other hand, fluticasone led to a 12% increase in the frequency of exacerbations treated by antibiotics alone, compared with placebo. Data from the Effect of Indacaterol Glycopyrronium vs. Fluticasone Salmeterol on COPD exacerbations (FLAME) trial showed that the combination of a long-acting beta-agonist (indacaterol) with a long-acting antimuscarinic (glycopyrronium), compared with the combination of a long-acting beta-agonist (salmeterol) and an ICS (fluticasone propionate) significantly reduced the rate of moderate or severe exacerbations treated with antibiotics and those treated with antibiotics and systemic corticosteroids.^104^ The impact of the two interventions on exacerbations treated with systemic corticosteroids alone was comparable.

These observations suggest that long-acting bronchodilators are effective in preventing all subtypes of exacerbations. On the other hand, ICSs may be more effective for people with frequent eosinophilic exacerbations but should be avoided in patients experiencing recurrent bacterial exacerbations. Further research is needed to validate these findings and evaluate the role of different medications in the exacerbations’ defined patient groups.

## The vision: personalised management of COPD exacerbations

Assessment, management and prevention of exacerbations are anticipated to change significantly in the future. As described, the heterogeneity in mechanisms and outcomes of COPD exacerbations can be resolved by grouping them aetiologically. Therefore, the main challenge to tackle is the development and validation of accurate biomarkers for early characterisation of the different types of exacerbations, which clearly extend beyond bacterial, viral and eosinophilic exacerbations that were discussed in detail here (figure 1). Such biomarkers could facilitate optimisation of exacerbation management and development of novel, targeted treatments. Since most exacerbations are addressed in primary care, the selected biomarkers will need to be measurable rapidly, near the patient, and simple, to facilitate implementation in primary care. RCTs focusing on the management of specific exacerbation subgroups are needed. These are likely to improve the clinical outcomes of exacerbations, but also COPD in general.
